# Implementation of patient participation in rehabilitation: An approach caught between different ideologies

**DOI:** 10.1177/13634593251374321

**Published:** 2025-10-04

**Authors:** Elin Margrethe Aasen, Marianne Kjelsvik, Lindis Katrine Helberget, Elisabeth Dahlborg

**Affiliations:** 1 Norwegian University of Science and Technology, Ålesund, Norway; 2 Faculty of Health Sciences, University West, Trollhättan, Sweden

**Keywords:** patient participation, rehabilitation, critical discourse analysis, person-centred, standardisation

## Abstract

The definition of specialised rehabilitation in Europe has changed from a focus on patients’ bodily functions and work tasks to a patient-centred focus prioritising patients’ wishes, allowing patients to actively collaborate and set their own goals. This study aimed to explore interprofessional healthcare teams’ discursive practice regarding the implementation of patient participation in specialised rehabilitation units in Norway. Data were collected from three focus groups with seven different health professions, totalling 18 healthcare professionals. A corpus-assisted critical discourse analysis outlined by Fairclough was used to analyse the data. Three interdiscursive discourses based on different and opposing ideologies were found: (1) the discourse of standardisation, in which healthcare professionals used international models for rehabilitation goal setting; (2) the discourse of interprofessional experts, in which healthcare professionals constructed themselves as experts; and (3) the discourse of patient responsibility, in which the patients were constructed as having rights and autonomy. The sociocultural practice of implementing patient participation in specialised rehabilitation in Norway highlighted a hegemonic struggle between standardisation; paternalistic and autonomy ideologies; ethical dilemmas between healthcare professionals’ knowledge and use of standardised goals; and patients’ autonomy, knowledge, and will.

## Introduction

Rehabilitation is a human right and critical intervention that enables people with disabilities to become independent. Independence entails full physical, social, mental, and vocational abilities, and full inclusion and participation in all aspects of life. Highly qualified healthcare professionals with shared professional values and ethical ideologies are prerequisites for achieving this goal (Hussein and Abou Hashish, 2023; Pakkanen et al., 2024; United Nations Commission on Human Rights, 1994).

A critical analysis of selected political documents on rehabilitation in Norway concluded that rehabilitation practices have become more interwoven with the roles of professionals, managers, and clients compared with the past (Feiring, 2012). The interdiscursive relationship between policy strategies and language use among Norwegian rehabilitation professionals is based on a combination of medical and psychosocial perspectives. These perspectives shape the discourses of rehabilitation as a clinical and management practice (Røberg et al., 2020). In the rehabilitation process, there has been a shift towards greater patient responsibility, more power to the patient (Negrini, 2018). Discourse analysis can uncover power exchange between healthcare personnel and the patient (Fairclough, 2010) and therefore, power is a key concept closely linked to patient participation (Aasen et al., 2012). This study explored how interprofessional healthcare teams discuss, the discursive praxis of patient participation in specialised rehabilitation units in Norway.

Over the years, the definition of specialised rehabilitation in Europe has changed from a focus on patients’ bodily functions and work tasks to a patient-centred focus where the patient’s wishes and priorities are central and they actively collaborate and set their own goals (Negrini, 2018). Definitions of rehabilitation have focused on patient-centred care; however, person-centred care is increasingly being advocated in rehabilitation settings (Lloyd et al., 2018). A change from patient- to person-centred care requires a shift in focus from functional life to meaningful life (Eklund et al., 2019). However, a systematic review found that rehabilitation practices have not yet fully implemented person-centred care (Yun and Choi, 2019).

**Figure 1.**
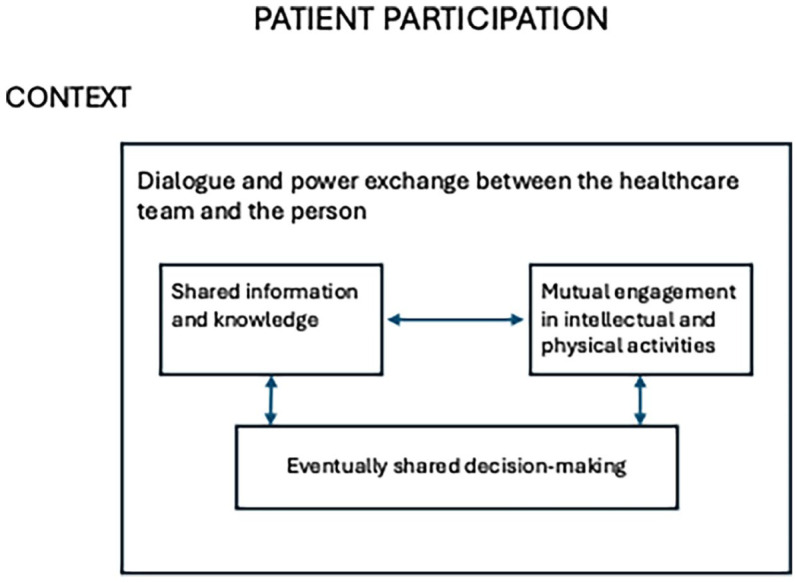
Patient participation. (Aasen, 2016) (p.156).

The concept of patient participation and similar concepts such as patient involvement and engagement have long been discussed; however, the differences are not distinct in the literature (Halabi et al., 2020). Our study was based on the operationalisation of patient participation given by Aasen et al. (2012, p.62) (Figure 1):

‘A process of power exchange between the healthcare team and the person. Participation does not necessarily require shared decision-making but a dialogue with shared information and knowledge and mutual engagement in intellectual and physical activities influenced by the context and characteristics of the individuals involved.’

A previous study described patients’ participation in specialised rehabilitation in Norway as being dependent on person-centred cultures and time. Patient participation changes throughout the rehabilitation process, and dialogue and power balance change from dependent to independent (Aasen et al., 2021). As autonomy increases, patients’ role in the rehabilitation process develops from passive to active (Proot et al., 2007). Rehabilitation is likely to be effective if it combines independence, autonomy, and social engagement in services to achieve a good quality of life (McClure and Leah, 2021). Patients’ participation in clinical decision-making indicates positive outcomes in rehabilitation; however, some patients have highlighted problems with information-sharing and felt that the staff might not listen to them (Rose et al., 2019). Healthcare professionals and patients may hold different attitudes towards patient involvement and different views on what it entails (Løwe et al., 2021). A study revealed that occupational therapists face challenges when it comes to setting limits and optimising patient participation in decision-making, avoiding ethical dilemmas, and acting professionally (Kassberg and Skär, 2008).

The World Health Organization developed a framework for measuring health and disability at individual and population levels in the International Classification of Functioning, Disability, and Health (ICF). The ICF has standardised functional areas such as cognition, language, mobility, and mental functioning (Kostanjsek, 2011; World Health Organization, 2001). This standardised framework is also used as a goal-setting tool for specialised rehabilitation in Norway (Norwegian Association for Physical Medicine and Rehabilitation, 2012). Studies have shown that goal-setting meetings in rehabilitation are important forums for patient participation (Rose et al., 2017, 2019).

Barnard et al. (2010) emphasised that there is no straightforward translation of patient wishes into agreed-upon written goals, and that patients and healthcare professionals may have different rehabilitation goals. Maribo et al. (2020) pointed out that while patients relate their goals to returning to everyday life, healthcare professionals tend to be more oriented towards physical and cognitive rehabilitation. The European Standard on Patient Involvement in Healthcare (Stjernswärd and Glasdam, 2022) highlights the need for further research on the empirical applications of patient participation.

## Aim

To explore interprofessional healthcare teams’ discursive practices regarding the implementation of patient participation in specialised rehabilitation units in Norway.

## Methodology

### Research design

Patient participation concerns the power order between patients and healthcare professionals, and critical discourse analysis (CDA) enables us to uncover what this power order looks like. We conducted Fairclough’s (2010) CDA to describe, interpret, and explain the data, which was supported by corpus-assisted CDA (Crawford et al., 2013, 2014).

The starting point of discourse analysis research is the recognition that objectivity cannot be fully attained. This acknowledgement arises from the understanding that the reality we study is socially constructed and rooted in sets of assumptions, some of which may remain imperceptible (Dahlborg et al., 2024).

Discourse analysis focuses on language. According to Fairclough, it is possible to identify different discourses using CDA. Discourse is a way of representing certain parts or aspects of the world that represent social groups and relations between social groups, that is, different institutions in society in different ways (Fairclough, 2010).

The order of a discourse can be viewed as a combination of different distinctively articulated discourses (Fairclough, 2010). Therefore, the order of a discourse encompasses a discursive practice in which there is a struggle for ideological hegemony, implying that discursive practices can be reproduced or replaced, thereby altering the prevailing order. Discourse and any specific instance of discursive practice can be observed simultaneously in language text (spoken or written) and discourse practice (e.g. text production and interpretation). Additionally, they are situated within the sociocultural practice, with a piece of discourse embedded at various levels, including the immediate situation, broader institution or organisation, and societal level (Fairclough, 2010).

The central concepts related to CDA are ideology, hegemony, and power. Fairclough defined ideology as a set of beliefs and attitudes, whereas hegemony occurs when the discourse and the ideology to which it is connected dominate the discourse order. The hegemonic discourse, how the health personnel talk, has the power to affect practice. One purpose of discourse analysis is ‘to identify what discourse in a text is supported by societal institutions; therefore, it is allowed to make a cultural impact’ (Dahlborg et al., 2024). Clarifying the discourse order of patient participation in rehabilitation can deepen our understanding of the factors that affect the rehabilitation culture.

### Research context and participants

This study was conducted in two physical and rehabilitation medicine units in one Norwegian health region. Rehabilitation units are part of specialist healthcare services where interprofessional teams comprising physicians, nurses, psychologists, physiotherapists, speech therapists, social educators, occupational therapists, and social workers work. These units serve patients with moderate to severe difficulties and require special expertise in assessment and intervention. They have areas for examination, training, meals, and other activities (Directorate of Health, 2022).

A purposeful sampling strategy plan (Cheek and Øby, 2023) was developed to gain insight into various professionals’ experiences of patient participation. The contact persons in the two rehabilitation units informed all relevant professionals about the study and invited them to participate in a focus group interview. The inclusion criteria was that they worked clinically and had first-hand experience with the implementation of patient participation in specialised rehabilitation. The motivation to participate was high in both units. The sample included personnel across different ages, most of whom had several years of experience in long-term rehabilitation (Table 1). All participants were Norwegian-speaking and well acquainted with the Norwegian healthcare system. Representatives from all professional groups employed in rehabilitation departments participated in the study. Although the physicians held overall responsibility within the department, they participated equally with other members in the focus group interviews.

**Table 1.** Participants and settings in focus group interviews.

Group no.	No. of participants	Female/Male	Age mean (range)	Years of experience in specialised rehabilitation units mean (range)	Interprofessional focus groups consisting of physicians (PH), nurses (RN), psychologists (PS), physiotherapists (PT), speech therapists (ST), occupational therapists (OT), social educators (SE), and social workers (SW)
1	7	7/0	39 (24–47)	9 (1–20)	PH, PS, OT, PT (*n* = 2), RN (*n* = 2)
2	4	4/0	52 (41–63)	15 (3–31)	PH, RN, SW, PT
3	7	5/2	47 (38–60)	15 (1–34)	PH, SE, SW, OT, RN, PT, ST

### Data collection

Data were collected through three focus group interviews with 18 healthcare professionals working in specialised rehabilitation units in Norway. Focus groups were chosen as a valuable approach for capturing interprofessional healthcare group attitudes and norms according to patient participation, which might not be obtained from individual interviews. Purposive sampling (Krueger and Casey, 2015) was used, with the goal of representing all professions in the units in each focus group. The focus group interviews were conducted over two months in 2020 in sheltered rooms at healthcare personnel workplaces, lasted between 35 and 100 minutes, and was audio recorded. In all interviews, the first author (female, PhD) was the moderator, and the second author (female, PhD) was the co-moderator, both of whom were experienced and interested in the topic. Before data collection, the researchers had no prior knowledge of the participants.

In the focus groups, the healthcare professionals were asked to state and discuss how they collaborated with the patients in the rehabilitation process and the primary health services provided from the day the patient arrived until they left and returned home. The moderator’s role was to facilitate natural, easy conversations between the group participants, and encourage them to elaborate and give examples (Krueger and Casey, 2015). The healthcare professionals provided detailed descriptions and presented open and shared attitudes regarding their experiences with patient participation in the rehabilitation process. The moderators arranged for everyone to participate in the conversation and share their experiences. The co-moderator observed the group interactions, took notes, and asked follow-up questions. The second author transcribed and depersonalised the interviews verbatim, then the authors checked the transcriptions for accuracy. All the interviews were conducted and transcribed in Norwegian.

### Data analysis

To analyse the text (corpus), we used a corpus analysis (Anthony, 2010) to establish the quantified ‘linguistic fingerprint’ (Atkins and Harvey, 2010) of the transcribed interview, followed by a CDA to describe, interpret, and explain the data (Fairclough, 2010) as a three-step process. We analysed the text in Norwegian and then translated the findings into English.

### Description of the text

**Figure 2.**
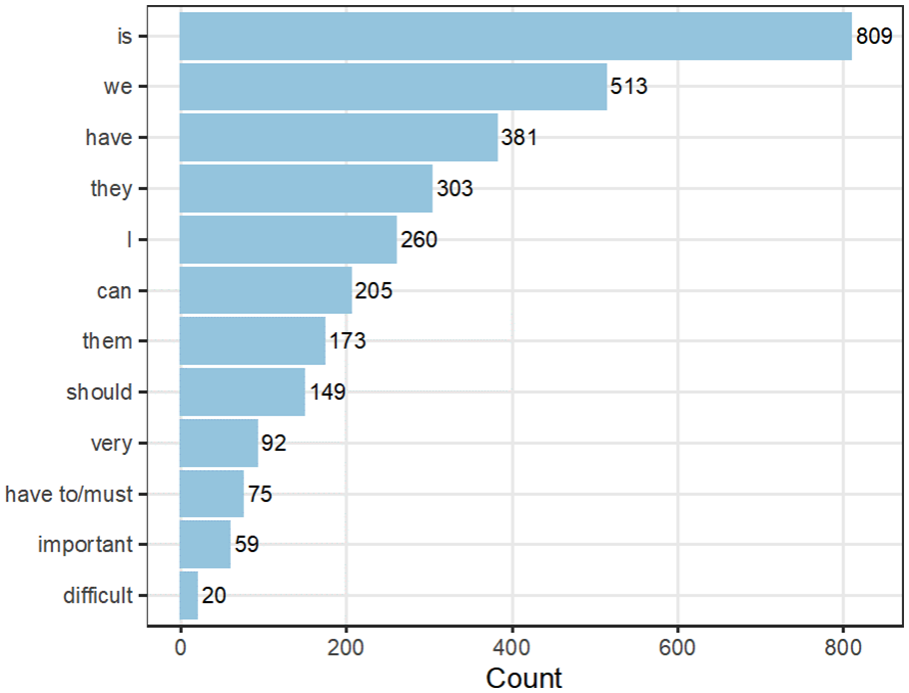
Sample word frequencies (total words = 24,770).

AntConc software (Anthony, 2010) was used to capture how words and grammatical formulations can be involved in the construction of discourses. This provided an overview or baseline of the wording and grammatical content of the text and the frequency of words and their combinations (collocations) to identify the ‘linguistic fingerprint’ or ‘aboutness’ of the text (Atkins and Harvey, 2010) (Figure 2 and Table 2). Textual analysis was used to capture how words and grammatical formulations participate in the construction of discourses. The modality of a sentence expresses the power level of the interaction between patients and healthy individuals. This process involves the use of evaluative words, personal pronouns (instead of names), modal auxiliary verbs (will, can, should, and must), and active and passive words. We examined the frequency of words used in the interviews and combined the analyses of word frequencies and concordance lines (Figure 2 and Table 2) to achieve a broad ‘linguistic signature’ for the combined text, which supported our CDA.

**Table 2.** Concordance lines.

Is What *is* important for you. What *is* important for the patient. What is important for this person. It *is* important to explain along the way. It *is* important to have a conversation together. That one must be clear about it, what *is* our job and our professionalism. It is also the goal that he should be self-sufficient and that is the best.
We And *we* have conversations in the team. Then, *we* try to facilitate that also. *We* want them (patients) to be active. *We* saw clearly how the patients reacted positively. And then, *we* have board meetings every morning, and the patients are not there.
Have We *have* a structure. We *have* guidelines, and we *have* patient care with a focus on patient participation. We *have* read the referral before they arrive. We *have* already started the planning of their stay before they arrive.
They/them Then, *they* get to decide a little. *They*, the whole family is affected. As we get to know *them* better, it’s easier to figure out what’s important. It’s a bit forced, but it’s trying to get *them* involved in a good way.
I *I* think it has a bit to do with health professionals’ professionalism as well. *I* think one must be very clear on what is a professional initiative and what is something the patients can take responsibility for.
Can But you *cannot* do anything, you *cannot* control the patient. So, there *can* be other things that are more important than what one thought before. But then, there *can* be patients who do not want it.
Should How much can we limit, and how much *should* we adhere to their own wishes because it is difficult. It’s easy to say you *should* not do this, but it’s their choice after all.
Very They are *very* active, or we want them to be *very* active.
Have to/must I think we *must* listen to where the patient is. We may *have to* try to set some sub-goals, get them involved. We *must* push a little, then we *must* focus on what is close to the individual. We *must* respect their choice, but it is a dilemma.
Important It is *important* that this person feels he is being seen. It is *important* that they get to be part of the decision. It is *important* to follow up on what the patient’s goal and wishes are. Then, it is *important* to start a dialogue. So, maybe conversation is more *important* than testing.
Difficult It is *difficult* to mobilise them because they prefer to rest and are always tired.

### Interpretation of the discursive practice

The analysis of the discursive practice level involved scrutinising the text production processes as well as intertextuality and interdiscursivity, which refer to the text’s relationships with other texts and discourses. We looked for words that originated from other discourses and understood how the words were used.

### Interpretation of the sociocultural practice

The analysis of the third dimension, sociocultural practice, identified the possible connections between discursive practices and societies. This also involved analysing whether and how sociocultural approaches can challenge the existing order of discourse. The interpretation of the sociocultural practice is presented in the Discussion section.

### Ethical considerations

The Regional Committee for Medical Research Ethics of Central Norway approved the study, and the Norwegian Centre for Research Data permitted the collection of the research data (ref: 2017/2290/REK midt and ref: 466052).

The managers of the participating units approved the study. The healthcare professionals who attended the focus group meetings received oral and written information about the aim of the study. Furthermore, they were assured that their participation was voluntary and that they could withdraw without having to explain their reasons. Written informed consent was obtained from all participants. The citing of quotations was related to the group and not individual participants to ensure anonymity during publication.

## Results

In the context of specialised rehabilitation in Norway, interprofessional healthcare professionals were invited to discuss how patient participation (Figure 1) was implemented. Three focus groups comprising 18 professionals were included in this study (Table 1). In the first instance, we examined the frequency of words used in the healthcare professional discussion (Figure 2 and Table 2) to achieve a broad ‘linguistic signature’ for the combined text to assist a critical discourse analysis.

The results demonstrated that patient participation had not yet been embedded in clinical practice. Analysis revealed three predominant discursive constructions influencing nursing care: the discourse of standardisation, which foregrounds protocol-driven approaches; the discourse of interprofessional expertise, where clinical authority is distributed among healthcare professionals; and the discourse of patient participation, which shifts emphasis toward patient autonomy and self-management.

How healthcare professionals used modal auxiliary verbs shifted from *we want* to *we should*, and *we are going to*, suggesting that patient participation has not yet been fully implemented in rehabilitation units’ culture.

*We have a structure, and we have guidelines, and we have patients who focus on patient participation. We should practice it (patient participation). We are going to make it (patient participation) together [. . .], and we are also going to make it entirely individually in the team.* (FG3)

The respondents also pointed to the specialised rehabilitation unit’s uniqueness in providing time for patients in the rehabilitation process. ‘We have a unique situation because we have more time with the patients here than in most regular hospital wards’ (FG3). Healthcare professionals’ narratives were constructed as three interdiscursive discourses: the discourses of standardisation, interprofessional experts, and patient responsibility.

### Discourse of standardisation

Healthcare professionals discussed how to plan patient rehabilitation. The team prepared itself based on the written information from the referring doctor before the patient arrived at the unit. Healthcare professionals constructed a structure in the units based on international guidelines. International models for rehabilitation goal setting (ICF) were used, and healthcare professionals described how they discussed the goals with the patients. However, prior to goal setting, the patients were not asked about their wishes and knowledge.

*When the patient arrives at the unit, they first meet a doctor who runs a physical examination, and then all professional groups make their assessments. During the first week, we have a joint meeting with the team, a mapping meeting where we use the ICF model for mapping.* (FG2)

The healthcare professionals classified the patients according to the ICF standardised functional areas, such as cognition, language, mobility, and mental functioning ‘The model focuses on the patient’s physical level’ (FG2).

After the healthcare professionals had classified the patients, they called the patients to join the meeting, and the healthcare professionals had a dialogue with the patients and agreed on the plan for the patient’s rehabilitation. The meeting for goal setting at which the patient was present was scheduled for the first week or the week after the patient’s arrival. ‘The patient should set goals for the rehabilitation stay, and the relatives are also often invited to attend those meetings’ (FG1).

This plan included the main goals and sub-goals and identified the personnel responsible for conducting the training. The plan was stored in patient rooms and evaluated in collaboration with the patients after 14 days. Additionally, the healthcare professionals held blackboard meetings every morning during the stay without the patient to discuss the patient’s achievement of goals.

The discourse of standardisation centred on the ways in which healthcare professionals classified patients using the standardised functional domains outlined by the International Classification of Functioning, Disability and Health (ICF), both prior to and following direct patient encounters

### Discourse of interprofessional experts

The interprofessional expert discourse constructs the healthcare professionals as experts in rehabilitation and patient participation. The respondents noted the importance of working and collaborating well with interprofessional teams. They used the word ‘we’ when discussing the interprofessional healthcare team. It was one of the most frequently used words in the interviews (Figure 2 and Table 2). They pointed out that dialogue plays an important role in interprofessional teamwork.

*The structure is very flat; thus, it is not true that some sit much higher than others. It is nice to have an open dialogue, be a little critical of each other, and ask questions.* (FG1)

However, they never included patients in ‘*we*’; the dialogue occurred between professionals. Healthcare professionals had the power and claimed they knew what was best for the patients. ‘It is also the goal that he (the patient) should be self-sufficient, and that is the best’. This confirmed the healthcare professionals’ power and that they knew what they were talking about and doing. When healthcare professionals used the personal pronoun ‘I,’ they often used the word ‘think’ to express expertise: ‘I think one must be clear on a professional initiative and for what the patients can take responsibility’. When they talked about the patient, they used the words ‘they’ or ‘patient,’ often with ‘can’. They stated, ‘But you (healthcare professionals) cannot do anything. You cannot control the patient’. Healthcare professionals constructed themselves as powerless when they were unable to control their patients.

Healthcare professionals used the value word ‘challenging’ and the modal auxiliary verb ‘must’ when discussing their knowledge as healthcare personnel versus the patients’ wishes related to the rehabilitation process (Figure 2). In the interviews, healthcare professionals discussed the challenges that arose when patients wanted to do something with the risk of failing. ‘Furthermore, I think, we have some knowledge and experience of how things have gone in the past. . . [We] let them try it out. . . it is challenging’ (FG1). They used the word ‘responsibility’ when discussing their own and the patient’s responsibility:

*What is professional, what must we take responsibility for, what can the patient take responsibility for, what is our job and professionalism, and what is our ability to assess better than the patient?* (FG3)

The standardised goal of the ICF model was to focus on the functional level. However, not all patients managed their goals, and healthcare professionals had to consider what level of mastery was required for the patient. ‘I had to reduce those requirements because the requirements are often too high’ (FG2).

The healthcare professionals discussed what happens when patients return home and the **i**nterprofessional healthcare team no longer has the responsibility.

*So, that is a dilemma. In the municipality, they are allowed to stop walking and stop trying. What is a professional measure that we must take responsibility for, and what is something the patient can take responsibility for [. . .]? What is our job, what is our professionalism, and what is our ability to assess better than the patient?* (FG3)

The **i**nterprofessional healthcare team also constructed themselves as experts on patient participation:

*It is very much about how we, as staff, meet that user. Both with the words you choose and your body language [. . .] by behaving in a way that allows the user to feel safe, seen, and respected. You also experience that you must give them some time. It is a new life situation . . .. I think my role will also be to ask open questions, so that you can reflect a little about the patient’s needs and what the patient can be involved in.* (FG3)

After staying in the rehabilitation unit, the patients were able to evaluate their stay. ‘The feedback we get on the annual user surveys, or those sent out after they have left here, affects how we meet users in the next turn’ (FG3).

In the interprofessional expert discourse, the healthcare professionals were concerned about their professionalism and responsibilities.

### Discourse of patient responsibility

The respondents discussed how they collaborated with the patients and the importance of perceiving them as unique persons, as they were before the illness, with their own knowledge, will, and wishes but also responsibility. The discourse on patient responsibility was visible in how the healthcare personnel talked about the patient and used the words ‘must’ and ‘is’ and the value words ‘important’ and ‘difficult’ (Table 2).

*From the moment the patient enters the door, we begin to get the patient in focus and get to know them. ‘Who are you?’ ‘What has been important in your life?’ and ‘How is your situation now?’* (FG3)

Some patients were passive and did not want to exercise; the healthcare professionals had to push the patients and asked themselves who was responsible.

*You must put yourself in the harness to push, so that the patient does not feel they are being overrun. We want them to be active, but this is not always the case.* (FG2)

*How much can we limit and how much should we adhere to their wishes? It is difficult. It is easy to say you should not do this, but it is their choice after all.* (FG1)

The respondents used the words ‘difficult’ and ‘dilemma’ when discussing situations in which the patient’s will and wishes could harm their health. This was a dilemma between patients’ autonomy and healthcare professionals’ valuation of beneficence and no harm to patients' health.

*What is difficult is when the patient wants something that is harmful to their health. When the user participation principle clashes with health services and health promotion work . . .* W*hen patients are difficult to mobilise, they will not get up and will lie in bed all the time.* (FG3)

*I think one must be very clear on what a professional initiative is and what the patients can take responsibility for.* (FG2)

The discourse on patient’s responsibility focused on the patient’s autonomy, knowledge, wants, and will.

## Discussion

We explored the discursive practices of interprofessional healthcare teams regarding the implementation of patient participation in specialised rehabilitation units in Norway.

We found that the order of discourse for patient participation was a combination of three discourses: standardisation, interprofessional experts, and patient responsibility. The three discourses were based on different and opposing ideologies, beliefs, and attitudes (Fairclough, 2010), and ethical ideologies with different values and frameworks (Hussein and Abou Hashish, 2023). In this study, a hegemonic struggle was observed between standardisation, paternalistic and autonomy ideologies, and an ethical dilemma between what the patient can, and the healthcare professionals must take responsibility for.

When the healthcare team talked about patient participation, they often referred to goal-setting meetings and standardisation discourses. The patients’ goals were entered into the ICF goal system with standardised functional areas such as cognition, language, mobility, and mental functioning (Kostanjsek, 2011). A study from Denmark uncovered the ICF model as a biomedical discourse and a barrier to patient participation (Angel et al., 2023). In this study, the patients were not asked about their opinions until the healthcare personnel had set the goals. According to the definition of patient participation (Figure 1), shared information and knowledge are prerequisites for patient participation, and patients and healthcare professionals may have different rehabilitation goals (Barnard et al., 2010; Maribo et al., 2020). The challenge of presenting pre-defined goals while focusing on patient participation has also been highlighted by Oksavik et al. (2021). However, standardisation seems to be the answer when the World Health Organization and governments work on health service quality. Examples include international models for rehabilitation goal setting (ICF) (World Health Organization, 2001), the European Standard on Patient Involvement (Kostanjsek, 2011), and online information provided to cancer patients (Aasen et al. 2020). Perhaps the tension arising from international standards and prevailing rehabilitation culture may be impeding the implementation of person-centred approaches in rehabilitation care? If patients were actively involved from the outset of goal-setting processes, healthcare professionals could more effectively align care with individual needs and preferences. In this way, standardised goal-setting practices may be reframed as person-centred care, constituting a more responsive and individualized rehabilitation strategy.

The healthcare professionals in the present study emphasised the unique advantage of having more time with patients than in ordinary hospital wards. The importance of more time was also highlighted by patients in the Norwegian rehabilitation context in a previous study [name deleted to maintain the integrity of the review process]. Allowing patients to use the necessary time does not align with the findings of a CDA of Norwegian rehabilitation policies by Røberg et al. (2020). They found that individuals responsible for their health and well-being dominated the rehabilitation discourse. Service availability was constrained with shorter stays at the rehabilitation units. The power of healthcare professionals, the clinical practice experts, seemed to be weakened in Norwegian policy strategies. Owing to standardisation with pre-defined goals, healthcare professionals’ opportunities for action based on professional judgement may be limited. Simultaneously, patients’ ability to make decisions related to their health and rehabilitation is reduced.

Healthcare professionals did not talk about patients’ knowledge but about their own knowledge as experts in the interpersonal expert discourse. ‘Who has the main responsibility?’ was a key question for healthcare professionals when they believed that a patient’s actions could harm their health. Nevertheless, patient participation (Figure 1) demands a person-centred practice (European Committee for Standardization, 2020). Lloyd et al. (2018) argued that healthcare personnel must listen to the person and get to know ‘who they are’. However, paternalistic ideology is still reflected in documents such as the Patients’ and Service Users’ Rights Act in Norway (Aasen and Dahl, 2018). According to Fairclough (2010), discourses are in a dialectic relationship with sociocultural practice and not only influence sociocultural practice but are also influenced by it (Fairclough, 2010). Public documents with paternalistic and standardised discourses that govern healthcare may prevent healthcare personnel from adopting person-focused approaches. The White Book of Rehabilitation notes that rehabilitation should be patient-centred, with a focus on the patient’s functioning (Negrini, 2018), a biomedical ideology, ICF goals with standardised functional areas (Kostanjsek, 2011), and a standardisations ideology. A change from patient- to person-centred care requires a shift in focus from a functional life to a meaningful life for the patient (Eklund et al., 2019), entailing a humanistic ideology. Person-centred goal setting and care have proven to be possible in rehabilitation and can positively affect rehabilitation outcomes (Yun and Choi, 2019; Lloyd et al., 2018). The analysis in the present study showed that healthcare professionals suggested that patient participation and, thereby, a person-centred culture and humanistic ideology were not yet fully implemented in the specialised rehabilitation units.

Enabling patients to take responsibility for their rehabilitation process could present an ethical dilemma between the principles of autonomy and beneficence and no harm (Beauchamp and Childress, 2019). Patient participation is not only about patient autonomy but also requires dialogue with shared information and knowledge with mutual involvement in intellectual and/or physical activity (Figure 1). Gadow (1999) highlighted ethical narratives as emancipatory on the condition that they are jointly created by patients and healthcare professionals. If we adopt ethical narratives and change healthcare professionals’ question from ‘who has the main responsibility?’ to ‘what is the meaning and a good solution for the patients?’, the question of responsibility may not come up. According to Gadow (1999, p. 65), ‘because individuals are situated and self-interpreting, the most faithful way to know the good they seek is through their own accounts, their personal ethical narratives.’ To allow patients to participate (Figure 1) and shift the main goal of rehabilitation from a functional to a meaningful life (Eklund et al., 2019), patients’ ethical narratives (Gadow, 1999) and participation (Figure 1) may be an ethical ideology and framework for healthcare professionals (Hussein and Abou Hashish, 2023).

The order of the discourse of implementing patient participation in specialised rehabilitation seemed to involve an interdiscursive struggle between the discourses of standardisation, interprofessional healthcare experts, and the patient’s responsibility. These discourses in specialised rehabilitation can deepen our understanding of patient participation in a specialised rehabilitation culture as they arise at the intersection of the system, healthcare professionals, and patients.

## Strengths and limitations

We collected data through focus groups and asked an interprofessional team to narrate and discuss how they allowed patients to participate in the rehabilitation process. All interviews provided rich data and insights into the participants’ experiences related to patient participation. It was an engaging topic that offered everyone a chance to speak and reflect. Individuals from different professions had different perspectives and complemented each other, thereby strengthening the credibility of the study. Regarding the trustworthiness of the findings, the interviews might have lasted longer, thereby providing more examples of patient situations; however, on a busy clinical day, limited time was allocated for the interviews. Nevertheless, in accordance with the studied phenomena, discourses within rehabilitation settings provided sufficient data. A potential limitation of the study is the translation of results from Norwegian to English. However, particular care was taken throughout the process to ensure the integrity and accuracy of the original meaning. The strength of this study lies in the analysis; empirical discourse analysis can uncover the power dynamics between healthcare personnel and patients, and power is central to patient participation. The results can be generalised to similar specialised rehabilitation contexts in Norway.

## Conclusion

The sociocultural practice of implementing patient participation in specialised rehabilitation in Norway highlighted a hegemonic struggle between standardisation and the ideologies of paternalism and autonomy. The health personnel were uncertain about the rehabilitation process and what the patients could take responsibility for in the process. This challenge uncovered the ethical dilemmas between the international model for rehabilitation goal setting (ICF); healthcare personnel’s knowledge; and patients’ autonomy, wants, and will.

This study provides new insights that can be valuable for healthcare personnel and the government and may stimulate the discussion of standardisation versus patient participation. Furthermore, it can stimulate healthcare professionals’ discussions about their ethical frameworks.
